# Effects of Culture Systems and Nutrients on the Growth and Toxin Production of *Karenia selliformis*

**DOI:** 10.3390/toxins16120518

**Published:** 2024-12-01

**Authors:** Xizhen Wu, Guixiang Wang, Jiangbing Qiu, Aifeng Li, Philipp Hess

**Affiliations:** 1 College of Environmental Science and Engineering, Ocean University of China, Qingdao 266100, China; wuxizhen@stu.ouc.edu.cn (X.W.); wangguixiang@stu.ouc.edu.cn (G.W.); qiujiangbing@ouc.edu.cn (J.Q.); 2 Key Laboratory of Marine Environment and Ecology, Ocean University of China, Ministry of Education, Qingdao 266100, China; 3 Ifremer, PHYTOX Research Unit, F-44000 Nantes, France

**Keywords:** *Karenia selliformis*, gymnodimine A (GYM-A), culture systems, nutrients, toxin production

## Abstract

Harmful algal blooms (HABs) formed by toxic microalgae have seriously threatened marine ecosystems and food safety and security in recent years. Among them, *Karenia selliformis* has attracted the attention of scientists and society due to its acute and rapid neurotoxicity in mice. Herein, the growth and gymnodimine A (GYM-A) production of *K. selliformis* were investigated in diverse culture systems with different surface-to-volume (S/V) ratios and nitrogen/phosphorus concentrations. The results showed that the specific growth rates (μ), maximal cell yields, and GYM-A production levels of *K. selliformis* increased with higher S/V, but no significant differences were observed under different culture volumes with the same S/V, which indicated that light penetration and gas exchange in the seawater culture systems actually influenced the growth and GYM-A production of *K. selliformis*. The maximum cell density and photosynthetic efficiency of *K. selliformis* decreased under nitrogen (N) and phosphorus (P) deficiency, suggesting that the growth of *K. selliformis* was significantly inhibited by the deficiency in N or P. Both N and P limitation conditions, especially P deficiency, promoted the cellular GYM-A quotas of *K. selliformis*. In this study, a scientific basis is provided for understanding the effects of culture systems and nutrient concentrations on the growth and toxin production of *K. selliformis*.

## 1. Introduction

Harmful algal blooms (HABs), as an important marine environmental issue, frequently occur in marine ecosystems under global change conditions [1,2]. Diverse microalgal toxins can accumulate in marine organisms and thus enter human food chains [3]. Such toxins potentially cause poisoning events and also seriously threaten the health of marine ecosystems [4,5]. For example, the dinoflagellate *Karenia selliformis* formed blooms in various countries and regions around the world, causing large-scale fish mortality in some cases [6].

Gymnodimine A (GYM-A) was initially extracted from oysters (*Tiostrea chilensis*) collected from the South Island of New Zealand in 1994 and detected from the dinoflagellate *Gymnodinium* cf. *mikimotoi* [7]. Gymnodimines (GYMs) are known as “fast-acting toxins” due to their acute neurotoxicity to mice via intraperitoneal injection [8]. A variety of GYMs have been detected in shellfish samples from many countries and regions worldwide, including Europe [9,10], South Africa [11], North America [12], and China [13,14]. The dinoflagellate *K. selliformis* species, now included in the genus *Karenia* and which was previously described as belonging to the genus *Gymnodinium*, was identified as a GYM-producing microalga [15,16]. Subsequently, it was found in the coastal waters of New Zealand, Mexico, Kuwait, and Chile [16,17,18,19]. Recently, a large-scale bloom formed by *K. selliformis* and *K. mikimotoi* occurred in the waters of Southeastern Hokkaido, Japan [20].

The growth and toxin production of toxic microalgae are usually affected by a variety of factors. Physical factors, such as irradiation, temperature, and salinity, are important environmental factors affecting microalgal growth [21], while chemical factors such as inorganic salts, metal ions, and organic acids may significantly influence the intracellular toxin production of microalgae [22,23]. Studies on the effects of these physicochemical factors on the growth and toxin production of microalgae not only help in the prediction of blooms of harmful algae, but also improve the toxin yield from large-scale culture of microalgae in the laboratory [24,25].

In previous studies, higher maximum cell density and toxin production levels were observed when *K. selliformis* was cultured in smaller culture volumes [22,26,27]. However, it is still unknown how to explain this phenomenon. Some studies have shown that microalgae grow better in culture systems with larger ratios of surface to volume (S/V, cm^2^/L) [28,29]. The S/V ratios might be related to the air exchange rate of the culture system, which affects the CO_2_ concentration and the pH of the culture system [30].

Nitrogen (N) and phosphorus (P) are inextricably linked to growth and toxin production [31,32]. A 10-year field investigation of the dynamics of *K. selliformis* showed that the elevation of nitrate could promote the growth of *K. selliformis*, while the increase in total P would inhibit its growth of *K. selliformis* [33]. The current knowledge of the effects of N and P concentrations in culture media on *K. selliformis* is still too limited to model toxin production in laboratory conditions. Therefore, it is necessary to study the growth and toxin production of *K. selliformis* under N and P limitation conditions.

With the aim of obtaining larger quantities of toxins for the preparation of reference materials and toxicological studies, physical and chemical methods were adjusted and optimized to enhance the growth and toxin production levels of toxic microalgae [34]. However, the underlying factors for GYM-A production in different culture configurations are still unclear. Thus, the growth and GYM-A quotas of *K. selliformis* were investigated in culture systems with different S/V ratios and N and P concentrations in this study. The qualitative and quantitative analysis of GYM-A was carried out by the multiple reaction monitoring (MRM) mode of liquid chromatography coupled with quadrupole mass spectrometer (LC-MS/MS) [35].

## 2. Results

### 2.1. Effects of S/V on the Growth of K. selliformis

**Figure 1:**
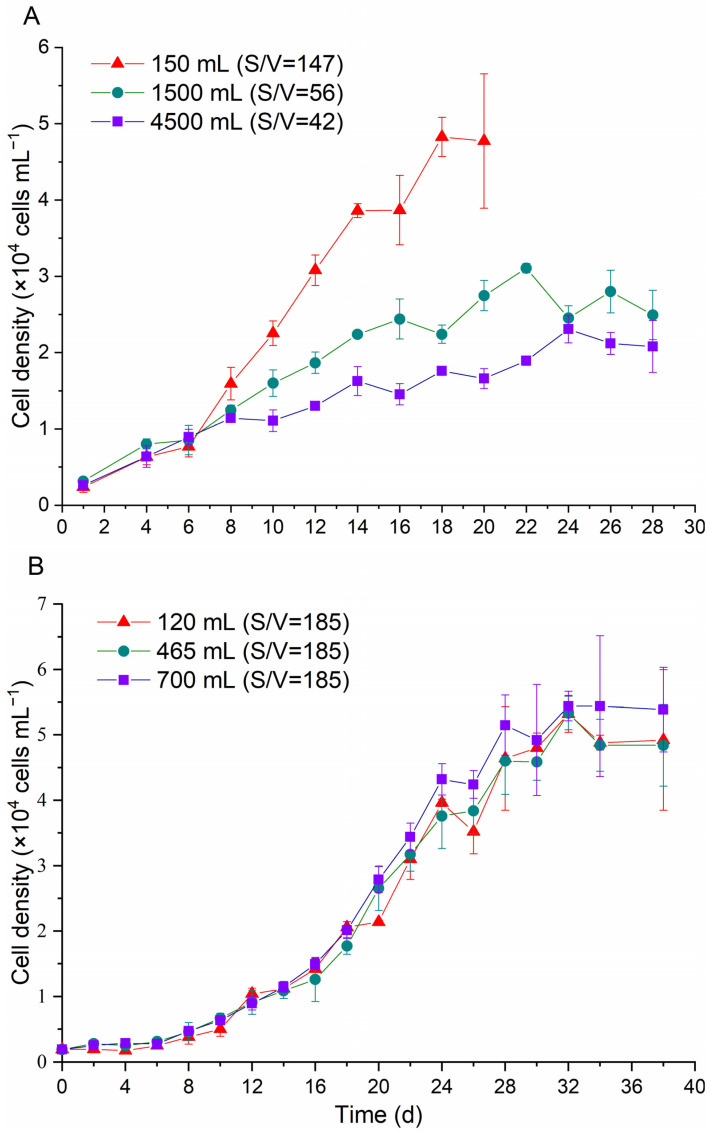
Growth curves of *Karenia selliformis* under different culture conditions. (**A**) Cultures in Erlenmeyer flasks with different surface-to-volume ratios (S/V, cm^2^/L); (**B**) cultures in different medium volumes with the same S/V ratios.

In the present study, the growth rates of *K. selliformis* increased with higher S/V values (Figure 1A), indicating that the S/V values were positively correlated with the growth of *K. selliformis*. For instance, the maximum cell yields of *K. selliformis* cultured in 150 mL culture medium (S/V = 147) were 2.1 times higher than that in 4500 mL (S/V = 42). The maximal cell yields and specific growth rates (μ) were significantly different (*p* < 0.05) in these three groups (Table 1), but no significant difference (*p* > 0.05) was observed in the growth of *K. selliformis* under different culture volumes when controlling the same S/V (Figure 1B).

### 2.2. Effects of S/V on the GYM-A Production of K. selliformis

**Figure 2:**
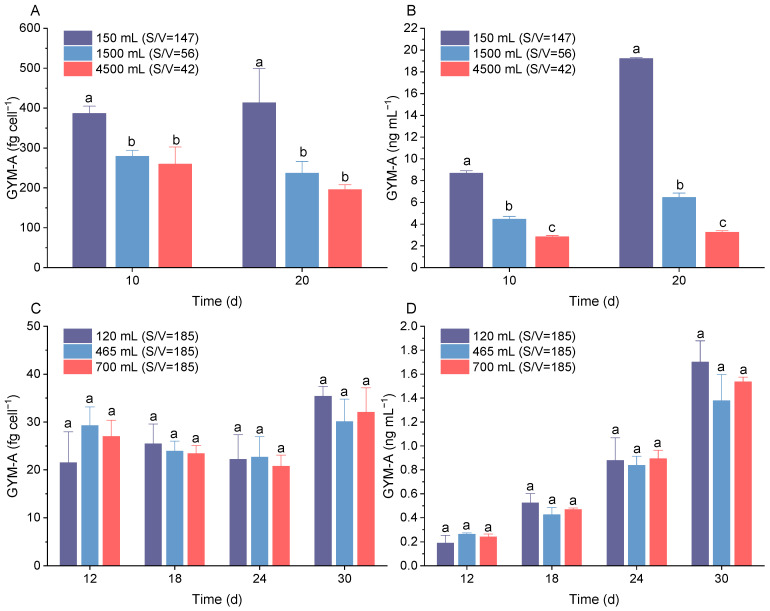
GYM-A quotas of *Karenia selliformis* under different culture conditions. (**A**) GYM-A yield (ng mL^−1^) and (**B**) GYM-A quota per cell (fg cell^−1^) of *K. selliformis* cultured in Erlenmeyer flasks with different surface-to-volume ratios (S/V, cm^2^/L); (**C**) GYM-A yield (ng mL^−1^) and (**D**) GYM-A quota per cell (fg cell^−1^) of *K. selliformis* cultured in different medium volumes with same S/V ratios. Treatments with different letters (a–c) are statistically significantly different (*p* < 0.05).

GYM-A was mainly detected in the strain of *K. selliformis* used in this study. The S/V ratios significantly affected not only the growth of *K. selliformis* but also GYM-A quotas. In this study, the cellular GYM-A quotas (fg cell^−1^) of *K. selliformis* increased with the increase in S/V values (Figure 2A). The GYM-A contents of *K. selliformis* in the 150 mL culture medium (S/V = 147) were significantly higher than that in the other two experimental groups (*p* < 0.05), in which the group of S/V = 147 yielded 2.1 times higher GYM-A cell contents than the group of S/V 42 at 20 days of incubation. In addition, the same trend was observed for the GYM-A concentration per culture volume (ng mL^−1^) (Figure 2B). Interestingly, when controlling the same S/V value, there was no significant difference in the cellular GYM-A contents of *K. selliformis* between different culture volumes (*p* > 0.05) (Figure 2C,D).

### 2.3. Effects of Nitrogen and Phosphorus on the Growth and Photosynthetic Parameters of K. selliformis

**Figure 3:**
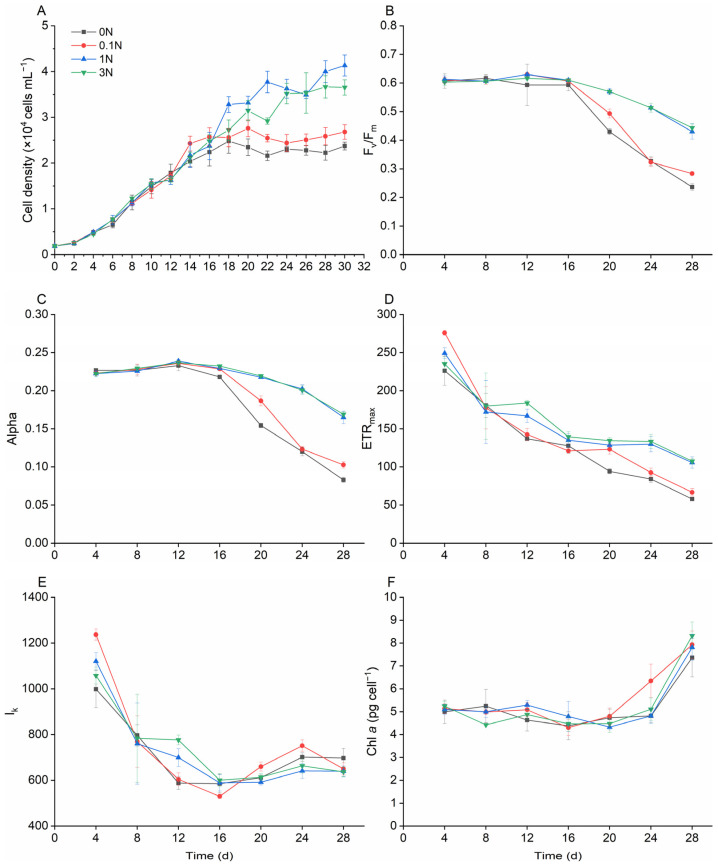
Effects of nitrogen concentration on the growth and photosynthetic parameters of *Karenia selliformis*. (**A**) growth curves; (**B**) F_v_/F_m_; (**C**) alpha; (**D**) ETR_max_; (**E**) I_k_; (**F**) *chl a* content per cell.

**Figure 4:**
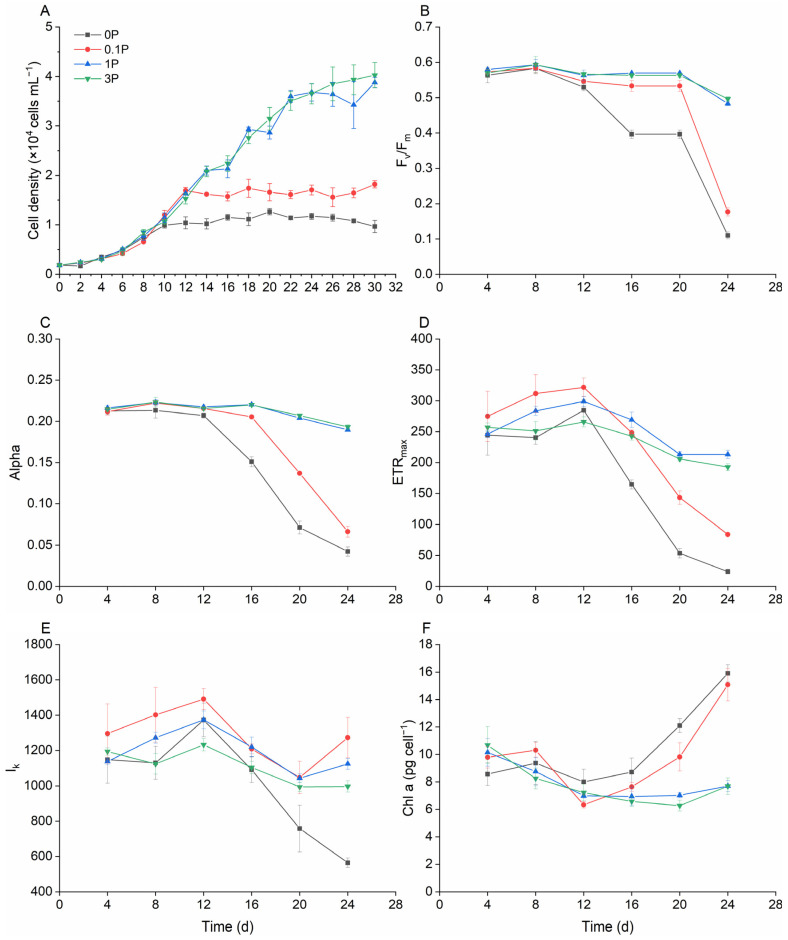
Effects of phosphorus concentration on the growth and photosynthetic parameters of *Karenia selliformis*. (**A**) growth curves; (**B**) F_v_/F_m_; (**C**) alpha; (**D**) ETR_max_; (**E**) I_k_; (**F**) *Chl a* content per cell.

Nitrogen (N) and phosphorus (P) are essential nutrients for the growth of phytoplankton; the lack and excess of either can affect the growth rate. In this study, the growth of *K. selliformis* was inhibited under N and P limitation (Figure 3A and Figure 4A). For example, the maximum cell density of the P-free group (0 P) reduced by 2.1-fold compared with that of the f/2 group (1 P) (Figure 4A). However, there was no significant difference (*p* > 0.05) between the growth condition of the N-rich group (3 N)/P-rich group (3 N) and that of the f/2 group (1 N/1 P), suggesting that excessive nitrogen and phosphorus content would not further promote the growth of *K. selliformis* (Figure 3A).

Photosynthetic parameters are closely related to the growth of phytoplankton. The results showed that F_v_/F_m_, Alpha, and ETR_max_ in the N-free group (0 N) and the N-deficient group (0.1 N) were significantly smaller than those in the f/2 group (1 N) (Figure 3B–D), a trend that coincided with the growth condition. However, no significant changes were found in I_k_ and the quota of chlorophyll content (Figure 3E,F). The values of F_v_/F_m_, Alpha, and ETR_max_ of *K. selliformis* also significantly decreased in P-deficient conditions (0 P and 0.1 P) (Figure 4B–D). Unlike in N-deficient conditions, the I_k_ showed a sharp reduction at the late growth stage in the P-free group.

The F_v_/F_m_-ratio is an important index characterizing the photosynthetic capacity of the phytoplankton photosystem II (PSII), which is the maximum photosynthetic yield of microalgae in darkness, and Alpha represents the light energy utilization efficiency of phytoplankton, The trends of both parameters were similar, which showed that the photosynthetic efficiency of *K. selliformis* decreased progressively with growth, while the low nitrogen content accelerated this response.

### 2.4. Effects of Nitrogen and Phosphorus on the GYM-A Production of K. selliformis

**Figure 5:**
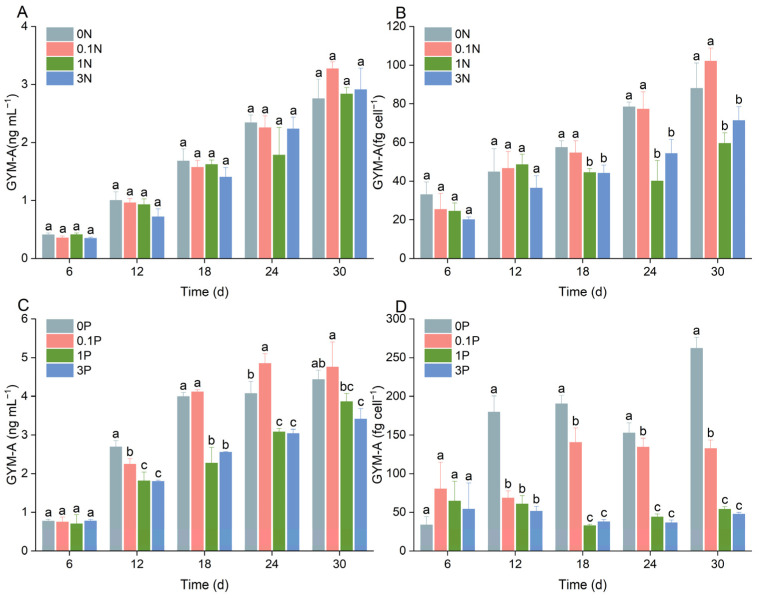
Effects of nitrogen and phosphorus concentrations on the GYM-A production of *Karenia selliformis*. (**A**) GYM-A yield (ng mL^−1^) and (**B**) GYM-A quota per cell (fg cell^−1^) of *K. selliformis* under different nitrogen concentrations; (**C**) GYM-A yield (ng mL^−1^) and (**D**) GYM-A quota per cell (fg cell^−1^) of *K. selliformis* under different phosphorus concentrations. Treatments with different letters (a–c) are statistically significantly different (*p* < 0.05).

In this study, it was found that both N and P limitation increased the GYM-A quota per cell of *K. selliformis*, with a larger increase being observed under P limitation. GYM-A quotas per cell under N limitation (0 N, 0.1 N) were significantly higher than that of the f/2 group (1 N) from day 18 (Figure 5A), whereas a significant increase appeared on day 12 under P limitation. Notably, the GYM-A quota in the P-free (0 P) group increased by 4.9-fold compared to that of the f/2 group (1 N) on day 30 (Figure 5C), indicating that the GYM-A synthesis of *K. selliformis* was strongly stimulated under P limitation. Additionally, the GYM-A yield (ng mL^−1^) did not significantly differ among the different nitrogen concentration groups (*p* > 0.05) (Figure 5B), while it was significantly enhanced by P limitation (0 P, 0.1 P, *p* < 0.05) (Figure 5D), indicating that the efficiency of GYM-A production by *K. selliformis* could be improved by P limitation.

## 3. Discussion

It has previously been shown that the S/V ratios of culture systems can influence phytoplankton growth [28]. The size and shape of culture vessels in laboratory can directly affect the growth of microalgae by altering light transmission and gas exchange at the interface of culture systems [36,37]. In the present study, the growth and GYM-A production levels of *K. selliformis* were significantly affected by the culture system volume when using different sizes of Erlenmeyer flasks with the same shapes in laboratory batch culture. The results showed that the growth rates and cellular GYM-A quota of *K. selliformis* increased when the seawater culture S/V increased. Beuzenberg et al., who cultured the *K. selliformis* using culture vessels of varying shapes, also found that the growth rates and toxin production levels of *K. selliformis* increased in smaller culture vessels [26]. The underlying reasons for these phenomena remain unclear. However, it has been demonstrated that the surface-to-volume (S/V) ratio of the culture system can influence the growth of microalgae [29].

The S/V of the culture system reflects the contact area per unit volume between the culture medium and the atmosphere, which can significantly influence the gas exchange between the culture system and the external environment [38]. Maximum biomass, volumetric, and area productivities of microalgae have been shown to increase with higher S/V culture systems [29]. Although different volumes of Erlenmeyer flasks have similar shapes, their S/V values are quite different. By comparing the S/V ratio of different culture systems in the current study, it was found that a higher S/V correlated with increased growth and GYM-A levels per cell. However, when the S/V values were maintained at a constant, variations in the volume of the culture systems did not result in significant changes in the growth and GYM-A production of the microalgae, indicating that the volume of the culture systems was not the direct cause of the changes in the growth and GYM-A production of the toxicogenic microalgae, and the microalgae did not have the ability to sense the variations in the culture system volumes.

The culture system with a larger S/V has more rapid gas exchange between the culture and the atmosphere [39]. In this study, the culture systems with the same S/V had a similar gas exchange despite the difference in culture volumes. Therefore, it can ensure that the culture systems had similar rates of CO_2_ uptake and release from the atmosphere, and were maintained in the same state of carbonate equilibrium. In addition, the irradiation was identical for all treatment groups with same S/V. The results of this study showed that the culture volume did not directly affect the growth and cellular GYM-A quotas of *K. selliformis*. However, we speculate that the different S/V ratios of Erlenmeyer flasks lead to differences in gas exchange, changing the growth and GYM-A production of *K. selliformis*.

The growth factors of microalgae were divided into resource factors and supportive factors (non-resources factors). The resource factors, such as nutrient elements, CO_2_, and sunlight, are directly utilized by microalgal cells, while the temperature and pH are supportive factors affecting microalgal metabolism [40]. Nitrogen (N) and phosphorus (P), as the essential nutrient elements, play an important role in the growth of microalgae. Feki et al. conducted a ten-year study of the Gulf of Gabes, and found that nitrate and total P levels significantly impacted the growth of *K. selliformis*. In the study, it was revealed that the elevated nitrate levels could promote the growth of *K. selliformis*, leading to increased production of GYM-A. Conversely, an increase in total P was found to inhibit the growth of *K. selliformis* in a long-term field survey [33]. However, no studies have been conducted under laboratory conditions to examine the effects of N and P on the growth and GYM-A production of *K. selliformis*.

Nitrogen is involved in the synthesis of different essential cellular components, such as proteins and chlorophyll, while P is a major component of membrane lipids, adenosine triphosphates (ATPs), and DNA [41,42]. Therefore, deficiencies and excesses of both nutrients can affect the physiological and metabolic processes in microalgae. In the current study, both the N and P limitation of *K. selliformis* reduced the maximum cell density and the photosynthetic efficiency, accompanied by an increase in cellular GYM-A quotas. The same trends were observed under N limitation in *K. brevis* [43]. In addition, the photosynthetic efficiency and chlorophyll metabolism of *Prorocentrum lima* were decreased following N deficiency [44]. Similarly, P deficiency led to suppressed growth and stimulated toxin production in the dinoflagellate of *Prorocentrum* spp. [45].

Nitrogen and phosphorus are crucial nutrients for carbon assimilation during photosynthesis [46]. Consequently, their deficiency inevitably impacts the photosynthetic parameters of microalgae. The limitation of both N and P can lead to a reduction in several photosynthetic parameters. For example, the F_v_/F_m_ of several typical marine microalgae significantly decreased under N and P limitation [47]. A similar trend was observed for *K. selliformis* in this study, suggesting that the photosynthetic efficiency of *K. selliformis* was significantly inhibited under conditions of N or P deficiency. These results provided strong evidence for the growth inhibition of *K. selliformis* under N or P deficiency. Interestingly, the chlorophyll *a* content significantly increased in the later growth stages of *K. selliformis* under P deficiency. A similar trend was observed in another dinoflagellate, e.g., *Amphidinium carterae*, where the cell cycle arrested in the G1 phase may be responsible for the excess accumulation of chlorophyll *a* [48].

Nitrogen and phosphorus limitations also remarkedly affected the toxin contents of cells. In this study, both N and P limitation could lead to a significant increase in the cellular GYM-A quotas of *K. selliformis*, which was consistent with observations on *K. brevis* in a previous study [43]. According to the carbon/nutrient balance hypothesis (CNBH), when the growth rate reduces under nutrient limitations (e.g., N and P), microalgae divert a greater portion of their fixed carbon from growth to defense, often in the form of increased production levels of allelopathic compounds (e.g., toxins) [49,50]. The results of the current study suggested that *K. selliformis* exhibited these same physiological patterns under N limitation in accordance with the CNBH. Additionally, the GYM-A yield of *K. selliformis* significantly increased under P deficiency, suggested that the efficiency of GYM-A acquisition can be improved by P limitation. Following this reasoning, N or P limitation in the marine environment may also enhance the ability of *K. selliformis* to produce GYMs, which may pose a more serious threat to marine ecosystems and human food safety and security. Marine regulators should pay more attention to N and P levels and ratios in marine environments to prevent or mitigate the negative impacts caused by toxicogenic microalgae.

## 4. Conclusions

In conclusion, in this study, the effects were investigated of culture systems and nutrient elements on the growth and toxin production of *K. selliformis*. Our findings demonstrated that the volume of the culture medium significantly affected the growth and GYM-A production of *K. selliformis*. However, the growth and GYM-A production of *K. selliformis* were not directly influenced by the volume of the medium, but rather by the varying S/V-ratios. Variations in the S/V of the culture systems likely affected the light penetration in seawater and the gas exchange at the interface of the culture system, leading to changes in photosynthesis and carbonate equilibrium, which in turn impacted the growth and cellular GYM-A contents of *K. selliformis*. Furthermore, we found that the growth and photosynthetic parameters of *K. selliformis* were inhibited under N and P limitation, while cellular GYM-A quotas were significantly enhanced. This suggested that N and P availability regulated growth by affecting photosynthetic efficiency, and that *K. selliformis* responded in particular to P limitation by increasing GYM-A production. These findings provided valuable insights into the formation of harmful algal blooms, with important implications for bloom prediction and marine ecosystem protection.

## 5. Materials and Methods

### 5.1. Chemicals

Acetonitrile and methanol with liquid chromatography (LC)-grade were obtained from Merck KGaA (Darmstadt, Germany). Ammonium hydroxide (NH_4_OH) was acquired from Fisher Scientific (Fair Lawn, NJ, USA), and dichloromethane was sourced from Macklin Ltd. (Shanghai, China). Certified reference material (CRM) of GYM-A was obtained from the National Research Council (Halifax, NS, Canada). Deionized water, with a resistivity of 18.2 MΩ cm or higher, was produced using a Milli-Q water purification system (Millipore Ltd., SAS, Molsheim, France).

### 5.2. Culture of Karenia selliformis

The monoclonal strain of *Karenia selliformis* (GM94GAB) isolated from the Gulf of Gabes, Tunisia [28] was provided by the PHYTOX Research Unit of Ifremer in Nantes. Referring to our previous study [36], the microalgae were cultivated in sterile natural seawater (121 °C for 20 min) filtered by a 0.45 μm mixed fiber filter (Xingya, Shanghai, China) and enriched with silicate-free f/2 medium. NaNO_3_ and NaH_2_PO_4_·H_2_O were added to the seawater medium as nitrogen and phosphorus nutrients. Cultures were maintained in an incubator at 18 °C with a constant top-light intensity of 6000 lux and a 12 h light: 12 h dark cycle. GYM-A dominated the toxin profile of *K. selliformis* and no other known toxins such as brevetoxin.

### 5.3. Record of Cell Density

A subsample of 1 mL from each culture was collected and fixed by Lugol’s iodine solution to determine cell density. Cell density was counted by light microscopy (Optika B-180; Italy) with a 0.1 mL phytoplankton counting chamber (DSJ-01; Xiamen Dengxun Equipment Co., Ltd.). Growth curves were drawn by the average cell counts from three replicates. Growth rates (*μ*) of *K. selliformis* were calculated according to the following formula:

*μ* = (ln*N*_t_-ln*N*_0_)/(*t*-*t*_0_)

where *N*_t_ and *N*_0_ are the cell densities at the time of *t* and *t*_0_ (d), respectively.

### 5.4. Experimental Design

#### 5.4.1. Comparison for Different Medium Volumes

Microalgae were cultured in conical flasks without agitation or air introduction in same shape with different surface-to-volume ratios (S/V, cm^2^/L), such as 250 mL conical flask with 150 mL media, 2000 mL conical flask with 1500 mL media, and 5000 mL conical flask with 4500 mL media. The surface areas and S/V ratios of different conical flasks are listed in Table 2. Cultures were incubated in the same incubator under identical conditions. Cell densities were recorded by light microscopy (Optika B-180; Italy) every two days. Cellular GYM-A quotas were monitored using 10 mL of culture collected on days 10 and 20.

Microalgae were also cultured in beakers with different volumes but the same S/V ratio. Since the shape of beakers is cylindrical, the same S/V can be obtained by maintaining a consistent liquid depth, such as a 150 mL beaker with 120 mL media, a 1000 mL beaker with 465 mL media, and a 2000 mL beaker with 700 mL media. The sides of the beakers were wrapped with aluminum foil to prevent side light, and sealed with transparent plastic wrap and perforated for ventilation. Beakers were randomly positioned in a top-light incubator to ensure exposure to equal light.

#### 5.4.2. Comparison for Different Nitrogen and Phosphorus Concentrations

Media containing different concentrations of nitrogen (N) and phosphorus (P) were obtained by adding different amounts of N and P to sterile natural seawater. The N and P concentrations of different treatments are listed in Table 3. The *K. selliformis* was cultivated in 500-mL conical flasks with 300 mL media, with an initial cell density of approximately 2000 cells L^−1^. Culture conditions were consistent with the experiments described above. Cell densities were recorded every two days, toxins were extracted every six days, and photosynthetic parameters were assessed every four days.

### 5.5. Measurement of Photosynthetic Parameters

Photosynthetic parameters of different N and P concentrations were analyzed using a phyto-pulse-amplitude-modulation (Phyto-PAM) chlorophyll fluorometer (Waltz, Germany) every four days. At each time point, 2 mL of culture was transferred to a quartz cup and dark-adapted for 10 min, then the initial fluorescence (F_0_) and the maximum fluorescence (F_m_) were measured. The maximum quantum yield of photosystem II (PSII) was reflected by the ratio of F_v_/F_m_, which was calculated as (F_m_-F_0_)/F_m_. The rapid light curves (RLC) were acquired with a total step number of 13, each lasting 20 s. Then, the photosynthetic parameters, including the initial slope of the curve (α, representing light utilization efficiency), maximum relative electron transport rate (ETR_max_), half-saturated light irradiance (I_k_), and chlorophyll a content, were acquired by the Phytowin V2.1.3 software.

### 5.6. Extraction of Toxins

To measure the contents of intracellular and extracellular toxins, 10 mL of culture were collected to extract GYM-A, according to our previous study with minor modification [51]. Briefly, cultures were centrifuged at 3000× *g* for 10 min at 4 °C, then the supernatant and pellet were used to extract extracellular and intracellular toxins, respectively. For extracellular toxin extraction, 1 mL of dichloromethane was added the culture medium (10 mL). The samples were vortexed for 2 min and centrifuged at 5000× *g* for 5 min at 4 °C. The organic phase at the lower layer was collected and evaporated under a gentle stream of nitrogen. The residual material was reconstituted with 5 mL of methanol. Aliquots of samples (1 mL) were filtered (0.22 μm) and placed in a 1.5 mL glass vial for LC-MS/MS analysis. For intracellular toxin extraction, 2 mL of methanol were added to the pellet and then sonicated by a sonication probe with frequency at 20 kHz and amplitude at 55% (KS-750F; Kesheng Ultrasonic Equipment Ltd., Ningbo City, China) in an ice water bath for 5 min, and centrifuged at 5000× *g* for 5 min at 4 °C. The supernatant was transferred to a 5 mL volumetric flask. The extraction was repeated twice with an additional 2 mL of methanol. All the extracts were mixed and diluted with methanol to volume. Aliquots (1 mL) of samples were filtered (0.22 μm) and placed in a 1.5 mL glass vial for LC-MS/MS analysis.

### 5.7. LC-MS/MS Analysis Method

The contents of GYM-A in all samples were measured by an Ultimate 3000 HPLC system (Thermo Fisher Scientific, Bremen, Germany) coupled with an AB-Sciex Qtrap 4500 mass spectrometer (AB Sciex Pte. Ltd., Singapore) using an ESI interface. An X-Bridge™ C18 column (150 × 3 mm, 5 µm, Waters, Milford, MA, USA) was employed to separate toxins at 35 °C. The mobile phase was composed of solvent A (deionized water) and solvent B (90% acetonitrile), both containing 6.7 mmol L^−1^ NH_4_OH. A gradient elution was run at a flow rate of 300 µL min^−1^ from 50% to 100% B over 8 min, followed by a 1 min hold before return to 50% B with a 1 min re-equilibration period for each subsequent run. Injection volume was set at 5 µL, and multiple reaction monitoring (MRM) in positive mode was employed for the identification and quantification of GYM-A using three transitions (*m*/*z* 508.3 –> 490.3/392.2/162.1). The retention time of GYM-A was 7.57 min under the separation conditions.

### 5.8. Statistical Analysis

Statistical analyses were conducted with SPSS version 25.0 (IBM corporation, Armonk, NY, USA), where a one-way analysis of variance (ANOVA) with the least significant difference (LSD) post hoc test was employed to evaluate the significance of differences among the groups, with a predetermined significance level of *p* < 0.05. The software package of Origin 2022 (Origin Lab, Hampton, MA, USA) was utilized for data visualization.

## Figures and Tables

**Table 1:** The growth conditions of *Karenia selliformis* in treatments with different surface-to-volume ratios.

Medium Volume (mL)	Surface-To-Volume Ratio (cm^2^ L^−1^)	Maximum Cell Yield (cells mL^−1^)	μ (d^−1^)	Standard Deviation
150	147	48,260 ^a^	0.18 ^a^	0.003
1500	56	31,060 ^b^	0.11 ^b^	0.001
4500	42	23,060 ^c^	0.09 ^c^	0.003

Note: different letters show the significant differences, *p* < 0.05.

**Table 2:** The surface area and surface-to-volume ratios of different medium volumes.

Groups	Conical Flask Volume (mL)	Medium Volume (mL)	Surface Area (cm^2^)	Surface-To-Volume Ratio (cm^2^/L)
1	250	150	22.1	147
2	2000	1500	84.6	56
3	5000	4500	186.8	42

**Table 3:** The nitrogen and phosphorus concentrations in different treatments.

Treatments	N Contents (μmol L^−1^)	P Contents (μmol L^−1^)
0-N	0	36
0.1-N	88.2	36
1-N (f/2)	882	36
3-N	2647	36
0-P	882	0
0.1-P	882	3.6
1-P (f/2)	882	36
3-P	882	109

## Data Availability

The original contributions presented in this study are included in the article. Further inquiries can be directed to the corresponding authors.
